# Clinical performance of an atrial fibrillation burden tracking algorithm: Evaluation against reference public and clinical textile electrocardiographic datasets

**DOI:** 10.1016/j.hroo.2026.02.025

**Published:** 2026-03-06

**Authors:** Arezoo Karimizadeh, Amin Mahnam, Soosan Beheshti, Bastien Moineau, Milad Alizadeh-Meghrazi, Yaariv Khaykin

**Affiliations:** 1 Electrical, Computer, and Biomedical Engineering Department, Toronto Metropolitan University, Toronto, Ontario, Canada; 2 Myant Textile Computing Inc., Mississauga, Ontario, Canada; 3 Southlake Health, Newmarket, Ontario, Canada

**Keywords:** Atrial fibrillation, Textile ECG, Wearable device, Machine learning, AF burden monitoring, Long-term monitoring, Classification

## Abstract

**Background:**

Atrial fibrillation (AF), the most common sustained cardiac arrhythmia, increases risks of stroke, heart failure, and mortality. Short-term electrocardiographic (ECG) monitoring often misses paroxysmal or asymptomatic AF, underscoring the value of textile ECG platforms for continuous real-world rhythm assessment.

**Objective:**

To develop and clinically validate a lightweight, interpretable algorithm for AF detection and burden estimation using textile ECG recordings acquired during daily life.

**Methods:**

We developed Textile AF-Tracker (TAF-Tracker), an RR-interval–based machine learning pipeline using entropy, Lorenz-plot, statistical, and fragmentation features with ECG quality metrics to detect AF in 60-beat segments. Multiple classifiers (Random Forest, XGBoost, Support Vector Machine, logistic regression, and a threshold-based method) were trained on public AF datasets and long-term 3-lead textile ECG recordings (SKIIN™). Data were split by subject into training, validation, and test sets to ensure unseen data were tested.

**Results:**

Across public long-term AF datasets, Random Forest and XGBoost achieved 96%–99% accuracy, 95%–99% sensitivity, and 96%-99% specificity. In 14-day textile ECG recordings from 47 AF patients, XGBoost reached 98.4% accuracy (sensitivity 96.1%, specificity 98.7%). AF burden showed a median absolute error of 1.3% (interquartile range 0.7%–1.8%). In healthy and noise stress data, specificity remained ≥99%, even during activity.

**Conclusion:**

A lightweight RR-interval-based machine learning algorithm on textile ECG accurately detects AF and quantifies burden in long-term recordings with minimal error. Combined with a comfortable multi-lead textile platform, it provides a practical alternative to Holter monitors and implantable devices for continuous AF surveillance and treatment assessment.

**Key Findings**

▪ We developed a lightweight, rhythm-based algorithm enabling accurate detection and atrial fibrillation (AF) burden estimation using wearable textile electrocardiogram (ECG) recordings, supporting continuous and unobtrusive monitoring in real-world settings.
▪ The system achieved high diagnostic accuracy, with sensitivity and specificity exceeding 95%, confirming its clinical reliability for identifying AF episodes and normal rhythms.
▪ The algorithm demonstrated strong robustness to noise and motion artifacts, maintaining stable performance in both controlled clinical environments and ambulatory monitoring conditions.
▪ The integration of cloud-based analysis with textile ECG technology introduces a novel, scalable approach for continuous AF surveillance and early clinical intervention.

## Introduction

Atrial fibrillation (AF) is the most common sustained cardiac arrhythmia and is associated with increased risks of stroke, heart failure, and all-cause mortality. It affects approximately 10.5 million adults in the United States and over 8.8 million in Europe, with prevalence increasing steeply with age, from 0.26% in individuals aged <50 years to 18.29% in those aged 80–89 years and 24.58% in those aged ≥90 years.^1^

Following the COVID-19 pandemic, several large observational cohorts have demonstrated a 57%–83% higher incidence of newly-diagnosed AF among individuals with documented SARS-CoV-2 infection compared with non-infected controls, translating to an incident AF rate of approximately 2.2%–2.6% in the year after infection.^2^^,^^3^

For clinical diagnosis, AF should be documented on a 12-lead electrocardiogram (ECG) or rhythm strip lasting at least 30 seconds.^4^

### Long-term AF monitoring and AF burden

Short-term ECG recordings, including standard 12-lead ECGs and 24-hour Holter monitoring, may miss paroxysmal AF (PAF) or asymptomatic episodes.^5^^,^^6^ Long-term continuous monitoring increases the likelihood of detecting AF.^5^, ^6^, ^7^ In a modeling study, once-daily spot ECG measurements were estimated to miss AF in 65% of at-risk patients, whereas continuous monitoring over 2 weeks substantially improved detection.^8^

AF burden, commonly defined as the percentage of monitored time spent in AF, captures disease severity beyond the presence or absence of AF episodes. Higher AF burden is associated with increased risks of stroke, heart failure, and adverse cardiovascular outcomes, whereas lower AF burden is linked to improved prognosis.^9^ In heart failure populations, strategies that reduce AF burden, such as early rhythm control and catheter ablation, may decrease mortality and heart failure–related complications.^9^ Accurate, scalable tools to quantify AF burden over extended periods are therefore clinically important.

### Wearables for AF monitoring

Implantable cardiac monitors (ICMs) provide high diagnostic yield for AF over multi-year periods but require an invasive procedure and device costs that limit widespread deployment.^10^

Recent advances in wearables have enabled non-invasive monitoring using wrist-worn devices and smartphone-based technologies. Apple™ Watch (Apple, Cupertino, California) and Fitbit™ Watch (Google, Mountain View, California) devices have shown high diagnostic accuracy for AF detection, with reported sensitivities and specificities up to approximately 95% and positive predictive values approaching 98% in select populations.^11^, ^12^, ^13^ (new study comparing detection sensitivities and specificities of the wrist-worn technologies) However, these devices generally rely on intermittent photoplethysmography (PPG) or single-lead ECG recordings, lack continuous multi-lead ECG, and may exhibit reduced specificity during physical activity because of motion artifacts.^11^, ^12^, ^13^

Smartphone-based systems and handheld ECG devices, such as PulseSmart™ app, ECG Check case™, and the Kardia Mobile™ (Alivecor, Mountain View, California) can provide higher-quality ECG tracings but are typically used intermittently and are not optimized for continuous monitoring.^14^, ^15^, ^16^ Chest-attached ECG patches (eg, Zio Patch™ [iRhythm, San Francisco, California], VitalConnect™ [San Jose, California], BodyGuardian™ [Preventice Solutions, Eagan, Minnesota], BardyDx CAM™ [Bardy Diagnostics, Washington], BioTel Heart™ [BioTelemetry, Pennsylvania], MediBioSense™ [MediBioSense, Oxfordshire, United Kingdom]) extend monitoring durations to 7.5–30 days but can cause skin irritation, discomfort, or adherence issues with prolonged wear.^17^

Textile-integrated ECG systems (textile ECG) embed electrodes and leads into garments or bands using flexible, skin-friendly materials. These systems have demonstrated adequate ECG quality for clinical use and no significant skin irritation, making them attractive for long-term ambulatory monitoring.^18^, ^19^, ^20^ Textile ECGs can be worn during typical daily activities, including walking and running, but motion artifacts and variable skin contact introduce noise that complicates AF detection. Consequently, AF detection algorithms intended for textile ECG must be robust to noise and variable signal quality.

### AF detection from ECG: rhythm versus morphology

Most AF detection methods use either RR-interval-based rhythm irregularity, ECG morphology, or a combination. RR-based approaches quantify beat-to-beat variability and irregularity of RR intervals and can be applied irrespective of ECG lead configuration, provided reliable R-peak detection is available.^21^, ^22^, ^23^, ^24^, ^25^ Entropy-based features (eg, Shannon entropy, sample entropy) and related measures have shown high performance for RR-based AF detection.^22^, ^23^, ^24^^,^^26^, ^27^, ^28^, ^29^

Morphology-based methods focus on atrial electrograms and P-wave morphology, often using advanced signal processing or deep learning.^30^, ^31^, ^32^, ^33^, ^34^, ^35^, ^36^ However, low-amplitude P waves, dependence on specific ECG vectors, and irregular baseline activity in AF pose challenges for consistent P-wave detection. Furthermore, atrial signals may be obscured by noise in ambulatory recordings, and performance may converge with rhythm-based methods when morphology becomes unreliable.^37^

### Study aim

We sought to develop and clinically validate a wearable AF monitoring system that estimates daily AF burden using a lightweight, interpretable algorithm integrated into the SKIIN™ (Myant Inc., Toronto, Canada) textile-based ECG platform. The algorithm primarily analyzes RR-interval–derived rhythm features combined with signal quality metrics to detect AF episodes in long-term recordings. Its performance was evaluated on both public ECG databases and wearable textile ECG data collected in real-world conditions, with a focus on accuracy of AF burden estimation under noisy recording conditions. The novelty of this study lies in addressing the domain gap inherent to textile-based ECGs by assessing algorithm resilience to motion artifacts and noise profiles characteristic of dry textile electrodes, which differ from the gel-based clinical datasets typically used for model training. In this study, AF burden was quantified using R-R interval-based analysis, a validated approach for long-term monitoring. While morphological information is available, its integration is planned for future larger-scale studies.

## Methods

### Textile ECG device

The SKIIN device is an elastic chest band available in multiple sizes to accommodate different body types. This elastic, textile-based multichannel chest band incorporates knitted fabric electrodes for continuous ambulatory ECG monitoring. Unlike conventional adhesive electrodes or consumer-grade chest belts, the integrated textile sensors provide stable skin contact during daily activities while minimizing discomfort and skin irritation. The SKIIN chestband uses dry textile electrodes, which requires natural skin moist for high quality ECG recording. Perspiration generally ensures good signal quality and the band’s knitted structure is highly breathable, allowing excess sweat to evaporate efficiently, to ensure comfort. The system is Health Canada-cleared for medical-grade ambulatory ECG acquisition and is designed for extended wear. Participants wore the band continuously during the monitoring period, removing it only for showering or washing. The band is washable and was engineered to tolerate repeated wear and folding without degradation of signal quality. It records 3-channel medical-grade ECG at a sampling rate of 320 Hz that are approximating standard leads V4,V5, and V6 and transmits data to a paired smartphone via Bluetooth. On-board processing with the Myant R-peak Quality Algorithm (MRQA) identifies R-peaks and computes an ECG signal quality index.

The present study developed an AF detection and burden estimation algorithm (Textile AF-Tracker, TAF-Tracker) for integration into the SKIIN platform, with the goal of identifying AF episodes that might be missed by shorter conventional recordings and estimating AF burden over extended monitoring periods.

### Overall algorithm pipeline

The TAF-Tracker pipeline comprises 4 main components:

1. **Heart rate and quality extraction**
  R-peaks and a segment-level ECG quality metric are derived from each ECG channel using MRQA. R-peak timings are converted into RR intervals.
2. **Feature extraction**
  To maintain a lightweight implementation compatible with wearable devices, the algorithm uses an RR-based approach. Features are computed from non-overlapping segments of 60 consecutive RR intervals and include:
  • Entropy-based features: coefficient of sample entropy, Shannon entropy per R-peak, and its mean, median, and standard deviation per segment.
  • Lorenz (Poincaré) plot features: measures of RR-interval irregularity (AFEv, IrrEv, PACEv, OriginCount) and SD1/SD2 derived from the Poincaré plot.^38^
  • Statistical features: minimum RR, mean RR, standard deviation of RR, median heart rate, root mean square of successive differences, coefficient of variation, and standard error of the mean.^39^
  • Fragmentation measures: PIP, IALS, PSS, and PAS, reflecting fragmentation of sinus rhythm.^40^
  • Signal quality: segment-level ECG signal quality derived from MRQA.
  • Features are standardized using the mean and standard deviation computed from the training set.
3. **Exclusion of non-diagnostic segments**
  Non-diagnostic segments were removed before classification based on:
  • Missing data.
  • Poor signal quality (low MRQA index).
  • Very low heart rate (fewer than 7 R-peaks within 10 seconds).
  Subjects with >95% of segments labeled as non-diagnostic were excluded from AF burden analyses.
4. **Classification**
  We evaluated several classification strategies:
  • Threshold-based decision rules on select features
  • Logistic regression (LR)
  • Random Forest (RF)
  • Gradient boosting (XGBoost)
  • Support Vector Machine (SVM)
Hyperparameters were optimized by 5-fold cross-validation on the training set. Optimized parameters included the regularization strength (C) for LR and SVM; the number of trees (n_estimators) and maximum depth (max_depth) for RF and XGBoost; and kernel type for SVM.

### Data sources

To ensure robustness and generalizability, TAF-Tracker was trained and evaluated on multiple datasets, including public databases and proprietary textile ECG datasets. Atrial flutter (AFL) was labeled as AF for the purposes of classification and burden estimation.

### Public datasets:

• **MIT-BIH Atrial Fibrillation Database (Mit-AF)**: 10-hour, 2-channel ECG recordings from 23 subjects (sampling 250 Hz) with beat-by-beat and rhythm annotations.
• **MIT-BIH Noise Stress Test Database (Mit-NST)**: Twelve 30-minute ECG recordings from 2 subjects (360 Hz) with varying levels of baseline wander, muscle artifact, and electrode motion artifact.
• **MIT-BIH Long-Term Atrial Fibrillation Database (Mit-LTAF)**: 24-hour 2-channel ECG recordings from 84 subjects (128 Hz), with expert-annotated AF episodes and beat annotations.

### SKIIN datasets:

• **SKIIN TIT dataset (TXTITP):** 1-hour recordings from 56 healthy participants performing 5 standardized tasks: T1 – seated; T2 – standing; T3 – lying; T4 – walking; T5 – running, including postural transitions. T5 is notably more noise-prone due to motion artifacts.
• **SKIIN Long-Term AF dataset (SKIIN LTAF)**: 14-day continuous textile ECG recordings from 47 subjects, some of them with known AF. An experienced cardiac technician annotated AF episodes and other arrhythmias. An experienced cardiac technician annotated AF episodes and other arrhythmias. The study included participants across all SKIIN chestband sizes (2XS: 4.26%, XS: 2.13%, S: 6.38%, M: 23.40%, L:21.28%, XL: 10.64%, 2XL: 14.89%, 3XL: 17.02%), representing both men and women ( 38% female), allowing evaluation of device performance across a variety of body sizes and genders.

All textile data collection procedures complied with institutional and national research ethics guidelines. The SKIIN LTAF protocol was approved by the Southlake Regional Health Centre Research Ethics Board (SRHC REB #073-1819; initial approval November 25, 2019). Written informed consent was obtained from all participants prior to data collection.

Details of patient demographic information and AF subjects are shown in Table 1.

**Table 1:** Details of public and textile ECG datasets used in this study

Dataset	Total patients	Age (mean ± SD)	Sex (% male)	Monitoring duration (h)	AF patients
Mit_AF	23	64 ± 14	60% male	10 h	23
Mit_LTAF (Train/validation/Test)	84 (46/12/26)	66.9 ± 11.2	65% male	24 h	84
Mit_NST	2	60± 9	50% male	0.5 h	0
SKIIN TIT	56	35.2 ± 10.9	48% male	1 h	0
SKIIN LTAF	47	55.5 ± 15.0	62% male	14∗ (24 h)	5

ECG = electrocardiogram; LTAF = long-term atrial fibrillation.

### Segment-based AF labeling

For performance evaluation, recordings were divided into non-overlapping segments of 60 consecutive RR intervals. Segment labels were derived from expert rhythm annotations. A segment was labeled as AF if AF or AFL persisted for at least 30 seconds within the segment window; otherwise, it was labeled as non-AF.

### Training, validation, and test splits

Model training used Mit-LTAF as the primary development dataset. A subject-wise 55/15/30 split was applied to divide subjects into training, validation, and test sets, using a fixed random seed to ensure reproducibility. This yielded 46 subjects for training, 12 for validation, and 26 for testing, corresponding to 23,068 AF segments and 21,135 non-AF segments, a relatively balanced class distribution.

To assess generalization, trained models were further evaluated on 5 independent datasets: Mit-AF, Mit-LTAF (unseen test data from 26 subjects), Mit-NST, SKIIN TIT, and SKIIN LTAF.

### Feature selection

Feature importance was evaluated using multiple complementary selection methods (including variance-based selection, mutual information, recursive feature elimination, LASSO regularization, and tree-based importance), with variance-based selection ultimately used to define robust feature groups for the final real-time implementation. While detailed feature ranking was not the primary focus, these analyses ensured the stability, consistency, and computational efficiency of the models across different feature sets.

### Evaluation metrics

Segment-based performance

For segment-level AF detection, we computed:

• Accuracy
• Sensitivity (recall)
• Specificity
• Precision (positive predictive value, PPV)
• F1-score
• Matthews correlation coefficient (MCC)
• Performance was reported separately for each dataset.

### Burden estimation evaluation

AF burden was defined as the percentage of total diagnostic monitoring time classified as AF. To quantify the accuracy of AF burden estimates in long-term recordings, we used the absolute AF burden estimation error (%EAF), as proposed by prior work.^41^ For each subject, %EAF was calculated as the absolute difference between true and predicted AF burden, expressed as a percentage of total monitored time. We reported quartiles (Q1, median, Q3), maximum error, and mean error for each model and dataset. Performance metrics are reported at the daily level, which serves as a per-subject metric for single-day recordings while capturing the longitudinal day-to-day consistency required for multi-day assessments. This reporting structure ensures that both inter-subject and intra-subject variability are accounted for, providing a transparent evaluation of algorithmic reliability across all cohorts.

## Results

### Non-diagnostic segments exclusion

To evaluate the viability of real-time monitoring, we analyzed the impact of signal quality on non-diagnostic segment exclusion. While clinical datasets (Mit_AF, Mit_LTAF, Mit_NST) exhibited negligible heart-rate-related exclusions (<1%), textile-based datasets showed a marginal increase (1.0%–2.8%). Signal quality exclusions remained generally low (0%–6%); however, the textile-based SKIIN TIT dataset required significantly higher exclusions (8%–52%), particularly during vigorous activity (eg, running). Data attrition in the 14-day longitudinal study was primarily attributed to maintenance cycles (laundering/showering) and connectivity issues, variables that can be mitigated through enhanced participant training. These results demonstrate that when data continuity is maintained, the resulting signal integrity is sufficient for robust AFib detection.

### Segment-based AF detection results

Segment-wise classification performance for LR, RF, SVM, threshold-based classification, and XGBoost across Mit-LTAF (validation and test), Mit-AF, Mit-NST, and SKIIN TIT is summarized in Tables 2.

**Table 2:** Segment-based classification performance of AF detection for different models, LR, RF, SVM, a threshold-based method, and XGBoost

	Mit_LTAF(validation)						Mit_LTAF(test)						MIT_AF						Mit_NST		SKIIN TIT	
	acc	f1	mcc	prec	sens	spec	acc	f1	mcc	prec	sens	spec	acc	f1	mcc	prec	sens	spec	acc	spec	acc	spec
LR	98.1	97.6	96.1	96.0	99.3	97.5	94.5	94.8	89.0	94.4	95.1	93.9	95.5	93.8	90.3	**95.0**	92.6	97.2	97.6	97.6	98.0	98.0
RF	99.1	98.8	**98.0**	**98.0**	**99.5**	98.8	96.3	96.4	**92.6**	**97.1**	95.8	**96.8**	96.2	**94.9**	**91.9**	93.6	96.3	96.2	97.6	97.6	98.0	98.0
SVM	99.2	98.9	98.2	98.3	99.5	99.0	96.4	96.6	92.8	96.8	96.3	96.5	95.4	93.9	90.2	92.3	95.5	95.4	99.0	99.0	97.0	97.0
Threshold	66.2	31.1	22.4	68.7	20.1	94.4	55.5	31.1	21.8	81.3	19.2	95.2	69.9	33.3	33.2	89.8	20.4	98.6	81.7	81.7	100	100
XGBoost	**99.3**	**99.1**	**98.6**	**99.0**	99.3	**99.4**	**96.8**	**96.9**	**93.7**	**97.7**	96.2	**97.5**	95.4	93.8	**90.2**	92.2	95.6	95.3	**99.7**	**99.7**	97.0	97.0

Boldface indicates the minimum average burden estimation error.

LR = logistic regression; LTAF = long-term atrial fibrillation; RF = Random Forest; SVM = support vector machine; XGBoost = Gradient boosting.

Across the long-term AF dataset (Mit-LTAF), RF and XGBoost achieved the highest performance. On the validation set, RF attained an MCC of 98% with accuracy 99.1%, F1-score 98.8%, sensitivity 99.5%, and specificity 98.8%. On the test set, RF achieved MCC 92.6%, accuracy 96.3%, F1-score 96.4%, precision 97.1%, sensitivity 95.8%, and specificity 96.8%.

XGBoost demonstrated similarly strong performance, with slightly higher MCC values in both validation (0.98.6%) and test (93.7%) sets and balanced sensitivity/specificity exceeding 96% in most datasets. In Mit-AF, RF and XGBoost again performed comparably, with RF showing marginally higher MCC.

The SVM classifier also yielded high segment-level performance, but RF and XGBoost consistently ranked highest across databases. In contrast, the threshold-based method performed poorly, with MCC values near 0.22 on Mit-LTAF and high AF burden overestimation in long-term data, indicating that simple thresholding of individual features was inadequate for robust AF detection.

In the noise stress dataset (Mit-NST) and the healthy textile dataset (SKIIN TIT), XGBoost exhibited excellent specificity (up to 99.7%), suggesting strong robustness to noise and motion artifacts. RF performed similarly but with slightly lower specificity in the noisiest conditions.

### Burden-based AF detection result

AF burden estimation error statistics for all classifiers across Mit-LTAF (validation and test), Mit-AF, Mit-NST, and SKIIN TIT are summarized in Table 3.

**Table 3:** Burden estimation error statistical parameters for different models, LR, RF, SVM, a threshold-based method, and XGBoost

	Mit_LTAF(validation)						Mit_LTAF(test)				MIT_AF					Mit_NST					SKIIN TIT				
	Q1	med	Q3	max	Avg	Q1	med	Q3	max	Avg	Q1	med	Q3	max	Avg	Q1	med	Q3	max	Avg	Q1	med	Q3	max	Avg
LR	0.3	0.8	2.5	6.7	1.9	0.9	2.7	7.9	34.9	5.4	0.5	2.8	4.4	16.8	3.9	0.0	3.2	5.4	13.3	4.4	0.0	0.0	0.0	0.0	0.0
RF	0.1	0.4	1.4	4.8	1.0	0.1	0.4	2.3	33.4	3.9	0.2	0.4	4.3	20.5	**3.2**	0.0	2.7	6.5	6.7	3.2	0.0	0.0	0.0	0.0	0.0
SVM	0.3	0.5	1.1	3.5	0.8	0.5	1.1	2.8	29.7	3.5	0.3	1.4	4.7	22.6	3.9	0.0	0.0	3.3	8.1	2.3	0.0	0.0	10.0	20.0	6.7
Threshold	1.4	29.7	53.1	98.7	33.6	9.5	26.9	79.3	96.9	40.2	4.0	18.8	46.6	99.5	26.6	0.0	0.0	38.7	53.3	18.4	0.0	0.0	0.0	0.0	0.0
XGBoost	0.1	0.5	1.1	2.1	**0.7**	0.1	0.7	2.5	30.3	**3.2**	0.1	0.5	4.2	29.2	3.8	0.0	0.0	0.0	2.7	**0.5**	0.0	0.0	5.0	10.0	3.3

Boldface indicates the minimum average burden estimation error.

LR = logistic regression; LTAF = long-term atrial fibrillation; RF = Random Forest; SVM = support vector machine; XGBoost = Gradient boosting.

For long-term AF detection, XGBoost maintained the lowest average AF burden error (0.7% in Mit-LTAF validation; 3.2% in Mit-AF), supporting strong generalization across datasets. RF yielded comparable average errors (approximately 1.0% and 3.2%, respectively) and tended to minimize errors in normal or low-burden datasets (eg, SKIIN TIT, Mit-NST), where both RF and XGBoost reported negligible or zero AF burden.

The threshold-based method exhibited markedly higher AF burden errors (average errors >30% in Mit-LTAF sets), reinforcing its unsuitability for clinical burden estimation.

Overall, XGBoost offered the best compromise between low average burden error across all datasets and robustness to noise, whereas RF provided near-zero error in clean, non-AF datasets while still performing competitively in long-term AF recordings.

Clinical assessment of wearable textile ECG for AF monitoring

Segment-level performance of RF and XGBoost on the 14-day SKIIN LTAF dataset from 47 AF patients is reported in Table 4. RF achieved an accuracy of 97.6%, F1-score 89.6%, MCC 88.6%, precision 84.2%, sensitivity 95.8%, and specificity 97.8%. XGBoost further improved these metrics, with accuracy 98.4%, F1-score 92.8%, MCC 91.9%, precision 89.7%, sensitivity 96.1%, and specificity 98.7%.

**Table 4:** Segment-based classification performance for recorded textile ECG from multiple subjects

	SKIIN_LTAF_db					
	acc	f1	mcc	Prec	Sens	Spec
RF	**97.6**	89.6	88.6	84.2	**95.8**	**97.8**
XGBoost	**98.4**	92.8	91.9	89.7	**96.1**	**98.7**

ECG = electrocardiogram; LTAF = long-term atrial fibrillation.

AF burden estimation errors for SKIIN LTAF are summarized in Table 5. For RF, the median %EAF was 1.6% (Q1 1.2%, Q3 2.7%) with a mean error of 2.1% and maximum error of 4.0%. XGBoost yielded median %EAF 1.3% (Q1 0.7%, Q3 1.8%), mean error 1.44%, and maximum error 2.8%. These results indicate that, over 14-day continuous textile ECG recordings, the algorithm can estimate AF burden with a typical absolute error of approximately 1%–2%.

**Table 5:** Burden estimation error percentage(%EAF) computed for 14 days of recorded textile ECG

	SKIIN_LTAF_db					
	Q1	Q3	max	mean	median	min
RF	1.2	2.7	4.0	2.1	1.6	0.91
XGBoost	0.7	1.8	2.8	1.44	1.3	0.61

ECG = electrocardiogram; LTAF = long-term atrial fibrillation; max = maximum; min = minimum.

## Discussion

This study demonstrates that a lightweight, interpretable RR-based algorithm can achieve high-accuracy AF detection and AF burden estimation using textile ECG recordings acquired under real-world conditions. When evaluated against expert-annotated public datasets, the best-performing models (RF and XGBoost) achieved segment-level sensitivity and specificity greater than 95%. On long-term textile ECG recordings from AF patients, XGBoost maintained high sensitivity (>95%) and specificity (>93%) and delivered low AF burden estimation error (mean 3.8% on public long-term data and ∼1.4% on textile data).

Importantly, the algorithm remained robust in the presence of noise and motion artifacts typical of ambulatory use (eg, walking and running), with very low false-positive AF burden in healthy participants and noise stress datasets.

The proposed TAF-Tracker was compared with previously published RR-based and morphology-based approaches using long-term AF datasets (Table 6, not reproduced here). In general, the proposed method achieved lower or comparable AF burden estimation error than most RR-based algorithms (eg, ArNet, VGGNet) and outperformed several morphology-based deep learning models in the long-term AF test set, while maintaining a relatively simple feature set and moderate computational complexity.^39^^,^^41^, ^42^, ^43^, ^44^

**Table 6:** Average %EAF for LTAF(test) and Mit-AF dataset

Feature type		LTAF(test)	Mit-AF
RR-based	ArNet^39^	6.2	3.3
morphology-based	1D-CNN^42^	4.9	6.1
RR-based	VGGNet^43^	5.6	3.9
morphology-based	MT-DCNN^41^	2.8	1.7
morphology-based	MS-CNN^44^	4.3	5.4
RR-based	TAF-Track (Proposed method)	3.2	3.8

AF = atrial fibrillation; LTAF = long-term atrial fibrillation.

Deep convolutional and recurrent neural networks using raw ECG morphology can achieve high detection performance but often require substantial training data, graphics processing unit resources, and are less interpretable.^30^, ^31^, ^32^, ^33^, ^34^ Additionally, their structural complexity and memory requirements (often >500 Kb to several MBs) present significant computational burdens for real-time applications; In contrast, TAF-Tracker relies on physiologically interpretable RR-interval features (entropy, Lorenz-plot irregularity, fragmentation) and signal quality measures, and an optimized tree-based models and achieve a significantly lower footprint of 245.05 Kb with near-zero inference latency, which can be implemented efficiently on resource-constrained wearable platforms

Accurate estimation of AF burden has direct implications for patient management. Higher AF burden has been linked to increased risks of stroke, heart failure, and mortality and may influence decisions regarding anticoagulation, rhythm control strategies, and timing of repeat ablation.^9^ Reliable, patient-friendly tools for long-term AF burden assessment could support earlier identification of patients with subclinical or paroxysmal AF, refine stroke risk stratification, and help evaluate treatment response.

Textile ECG platforms offer several practical advantages compared with ICMs, adhesive patches, or intermittent wearables:

• **Noninvasive, comfortable long-term wear**: breathable, flexible electrodes integrated into clothing or chest bands may improve adherence and reduce skin irritation.^18^, ^19^, ^20^
• **Continuous multi-lead ECG**: the 3-channel configuration provides richer data than single-lead devices and may support future morphology-based algorithms and clinician over-reads.
• **Integration with daily life**: garments can be worn during routine activities, enabling continuous monitoring without stigmatizing or conspicuous hardware.

The demonstrated performance of TAF-Tracker suggests that textile ECG combined with robust RR-based AF detection can provide clinically meaningful AF burden estimates over multi-day or multi-week periods, potentially serving as an alternative or complement to conventional Holter monitoring and ICMs in selected populations.

The medical-grade multi-channel ECG used in this study addresses diagnostic limitations in wearable rhythm monitoring by facilitating expert review of comprehensive waveform data.^45^ Unlike consumer-grade wearables, the availability of full ECG waveforms enables sophisticated morphological analysis beyond basic heart rate metrics. This capability specifically overcomes the technical barrier regarding broad QRS complex detection previously noted in the literature.^45^

### Strengths and limitations

Strengths of this study include:

• Use of multiple independent datasets (public and textile) spanning a range of recording durations, signal qualities, and clinical contexts.
• Subject-wise data splits to avoid information leakage between training and test sets.
• Explicit evaluation of performance under noisy conditions and in healthy wearers performing daily activities.
• Focus on AF burden estimation, not only binary AF detection, aligning the algorithm with clinically relevant outcomes.
  Limitations include:
• AFL episodes were grouped with AF for classification and burden estimation. While clinically reasonable for many management decisions, future work could distinguish AF from AFL where detailed rhythm characterization is necessary.
• Annotation of the SKIIN LTAF dataset was performed by a trained cardiac technician; although consistent and expert, adjudication by multiple electrophysiologists could further strengthen reference labeling.
• The study focused on AF versus non-AF segmentation; other arrhythmias (eg, frequent ectopy, atrial tachycardia) were not specifically modeled and may impact performance in certain populations.
• Real-time, on-device performance and battery impact were not formally tested, although the design emphasized lightweight features and models suitable for deployment on wearable hardware.

### Future directions

Future studies with larger and more diverse cohorts, including patients with cryptogenic stroke, heart failure, and post-ablation populations, are warranted to confirm generalizability and to explore how AF burden derived from textile ECG influences clinical decision-making and outcomes.

In addition, incorporating selected morphology-based features (eg, P-wave and QRS characteristics) from the multi-lead textile ECG, as well as context-aware heuristics using activity or posture data, could further improve robustness and specificity, particularly in noisy or borderline cases.^46^ Finally, prospective clinical trials comparing textile-based AF burden monitoring with Holter monitors, ICMs, and other wearables would help define the optimal role of this technology in contemporary AF care pathways.

## Conclusion

This study provides the first clinical validation of an AF detection and AF burden estimation algorithm applied to long-term textile-based ECG recordings. The proposed TAF-Tracker, integrated with a 3-lead SKIIN chestband device, achieved high sensitivity and specificity for AF detection across multiple datasets and delivered low AF burden estimation error in 14-day wearable recordings.

By combining a comfortable, noninvasive textile ECG platform with a robust RR-based machine learning pipeline, this approach enables continuous, real-world AF monitoring that may enhance AF detection, refine AF burden assessment, and support timely therapeutic decision-making.

## Declaration regarding AI assistance

No generative AI or large language models were used for scientific content, data analysis, or experimental design; any language edits were limited to minor grammatical or clarity improvements.

## Disclosures

Drs Karimizadeh, Mahnam, and Alizadeh-Meghrazi are employees of Myant Corp. Dr Moineau was previously employed by Myant Corp. The remaining authors report no conflicts of interest relevant to this article.
